# Potential risks in using midodrine for persistent hypotension after cardiac surgery: a comparative cohort study

**DOI:** 10.1186/s13613-020-00737-w

**Published:** 2020-09-14

**Authors:** Jan-Alexis Tremblay, Philippe Laramée, Yoan Lamarche, André Denault, William Beaubien-Souligny, Anne-Julie Frenette, Loay Kontar, Karim Serri, Emmanuel Charbonney

**Affiliations:** 1 Critical Care, Université de Montréal, 2900 Boulevard Edouard-Montpetit, Montréal, QC H3T 1J4 Canada; 2 Emergency Medicine, Université de Montréal, 2900 Boulevard Edouard-Montpetit, Montréal, QC H3T 1J4 Canada; 3 Critical Care, Institut de Cardiologie de Montréal, 5000 Rue Bélanger, Montréal, QC H3T 1J4 Canada; 4 Cardiac Surgery, Institut de Cardiologie de Montréal, 5000 Rue Bélanger, Montréal, QC H3T 1J4 Canada; 5 Centre Hospitalier de L’Université de Montréal, 1051 Rue Sanguinet, Montréal, QC H3T 1J4 Canada; 6 Critical Care, Hôpital du Sacré-Cœur de Montréal, 5400 Boul Gouin O, Montréal, QC H3T 1J4 Canada

**Keywords:** Midodrine, Vasoplegia, Vasodilation, Hypotension, Cardiac surgery, Vasopressor

## Abstract

**Background:**

Persistent hypotension is a frequent complication after cardiac surgery with cardiopulmonary bypass (CPB). Midodrine, an orally administered alpha agonist, could potentially reduce intravenous vasopressor use and accelerate ICU discharge of otherwise stable patients. The main objective of this study was to explore the clinical impacts of administering midodrine in patients with persistent hypotension after CPB. Our hypothesis was that midodrine would safely accelerate ICU discharge and be associated with more days free from ICU at 30 days.

**Results:**

We performed a retrospective cohort study that included all consecutive patients having received midodrine while being on vasopressor support in the ICU within the first week after cardiac surgery with CPB, between January 2014 and January 2018 at the Montreal Heart Institute. A contemporary propensity score matched control group that included patients who presented similarly prolonged hypotension after cardiac surgery was formed.

After matching, 74 pairs of patients (1:1) fulfilled inclusion criteria for the study and control groups. Midodrine use was associated with fewer days free from ICU (25.8 [23.7–27.1] vs 27.2 [25.9–28] days, *p* = 0.002), higher mortality (10 (13.5%) vs 1 (1.4%), *p* = 0.036) and longer ICU length of stay (99 [68–146] vs 68 [48–99] hours, *p* = 0.001). There was no difference in length of intravenous vasopressors (63 [40–87] vs 44 [26–66] hours, *p* = 0.052), rate of ICU readmission (6 (8.1%) vs 2 (2.7%), *p* = 0.092) and occurrence of severe kidney injury (11 (14.9%) vs 10 (13.5%) patients, *p* = 0.462) between groups.

**Conclusion:**

The administration of midodrine for sustained hypotension after cardiac surgery with CPB was associated with fewer days free from ICU and higher mortality. Routine prescription of midodrine to hasten ICU discharge after cardiac surgery should be used with caution until further prospective studies are conducted.

## Introduction

Depending on the definition used, between 5 and 25% of patients undergoing cardiac surgery with cardiopulmonary bypass (CPB) will present sustained hypotension after surgery presumably attributed to loss of peripheral vascular resistance [1, 2]. This phenomenon is most probably multifactorial [3–5], with inflammatory [6, 7] and ischemia–reperfusion [8] insults at the forefront.

These patients, besides receiving more intravenous fluid and vasopressors after their surgery [9], stay longer in the intensive care unit (ICU) [4] and are at increased risk of kidney injury [10] and mortality [11, 12]. While strategies have emerged to prevent and address post-CPB vasodilation [11, 13], including refinement of surgical techniques as well as the development of more biocompatible CPB tubing, there is currently no specific treatment postoperatively which would reduce dependence on intravenous vasopressors and length of stay in the ICU.

Midodrine is an orally administered alpha agonist with predictable hemodynamic effects and an established safety profile outside of the ICU [14–21], although its safety for patients with intradialytic hypotension has been questioned [22]. Still, its characteristics make midodrine an attractive agent for ICU patients presenting prolonged hypotension attributed to vasodilation who are otherwise stable, and this particular use has been increasingly described in the last few years [23, 24]. Results from small cohort studies suggest that weaning of intravenous vasopressors is accelerated with midodrine administration, without significant side effect [25–28], although a recent meta-analysis has not confirmed such results [29]. Most patients in these cohorts presented with hypotension from sepsis and none had undergone cardiac surgery with CPB.

The use of midodrine in the context of post-CPB vasodilation represents an interesting approach, given the somewhat predictable temporal pattern of hypotension, relatively short length of stay and availability of hemodynamic monitoring. In an attempt to hasten weaning of intravenous vasopressors and accelerate ICU discharge after cardiac surgery, intensivists in our center have been increasingly using midodrine in patients with post-CPB vasodilation. The main objective of this study was to explore relevant clinical impacts and assess the safety profile of this strategy. Other objectives consisted of describing the clinical contexts in which this strategy was used as well as the observed prescription patterns. Our hypothesis was that midodrine would safely accelerate ICU discharge and be associated with more days free from ICU at 30 days.

## Methods

This is a single center retrospective cohort study in which we evaluated the clinical course of patients who received midodrine at least once in the ICU while on intravenous vasopressor after cardiac surgery with CPB at the Montreal Heart Institute. The study protocol was approved by our institutional review board (#2017–2285).

### Study design

The midodrine group included all consecutive patients having received midodrine within the first week (168 h) after cardiac surgery with CPB, between January 2014 and January 2018 at the 24-bed surgical ICU of the Montreal Heart Institute. In our institution, there is no protocol to guide the use of midodrine and its prescription is at the discretion of the ICU physician. The control group consisted of patients from two observational prospective studies [30, 31] that were done between 2015 and 2017 in the same institution, that included adult patients undergoing cardiac surgery with CPB. In both groups, we only included patients who had received intravenous vasopressors for at least 12 h after surgery (see Additional file 1: Figure S1).

In both groups, patients were excluded if they were already receiving midodrine before surgery, if they needed mechanical circulatory support before surgery, had emergency surgery, transplantation or had cirrhosis. Of note, 12 patients from these aforementioned observational studies had received midodrine after surgery and were thus already included in the midodrine study group.

In an effort to reduce selection bias between midodrine and control groups, a propensity score (PS) was built, computing relevant baseline and surgical characteristics (see Table 1)to reflect the probability of receiving midodrine after surgery. After PS computation, pairs of patients were matched in a 1:1 ratio (midodrine:control) without replacement and with a caliper of 0.2.

**Table 1:** Demographic and intra-operative characteristics

	Midodrine group	Control group	*p* value
*N*	74	74	
Age	68 [62–75]	65 [58–73.25]	0.184
Male	45 (60.8%)	47 (63.5%)	0.735
Type of surgery			0.220
Revascularization	38 (51.4%)	33 (44.6%)	
Valve surgery	17 (23.0%)	19 (25.7%)	
Combined	16 (21.6%)	22 (29.7%)	
Other	3 (4.1%)	0 (0%)	
Urgent surgery	15 (20.3%)	10 (13.5%)	0.273
Euroscore II	1.94 [1–2.91]	2.08 [1.31–4.0]	0.088
CPB duration (min)	77 [61–111]	93 [68–120]	0.067
Aortic cross-clamp time (min)	56 [40–82]	69 [40–91]	0.188
PRBC during surgery	0.41 ± 1.33	0.24 ± 0.37	0.21
Fluid balance during surgery (ml)	1037 [318–1850]	951 [595–1605]	0.907
Receiving ACEi or ARB	42 (56.8%)	50 (67.6%)	0.175

*ACEi* angiotensin conversion enzyme inhibitors, *ARB* angiotensin receptor blockers, *CPB* cardiopulmonary bypass, *PRBC* packed red blood cells

The prespecified primary outcome was the number of days alive and free from ICU at 30 days (ICU-free days), to take into account mortality and ICU readmission after initial discharge. Prespecified secondary outcomes included hospital mortality, readmission to the ICU after initial discharge, cumulative ICU length of stay, total time on intravenous vasopressor therapy and occurrence of severe acute kidney injury during the study period.

### Data collection

A retrospective chart review of all patients meeting the inclusion and exclusion criteria was undertaken. The data was collected on a pre-tested collection sheet and included baseline characteristics such as age, sex, Euroscore II [32], preoperative use of ACE inhibitors or angiotensin receptor antagonists, type of surgery, urgent status of surgery, CPB and aortic cross clamp duration, number of intra-operative red blood cells transfusion and intraoperative fluid balance in ml. Post-operative data were collected from the moment of ICU arrival ending at hospital discharge or death. Doses of norepinephrine as well as other vasopressors (vasopressin, phenylephrine, epinephrine) and inotropes (dobutamine, milrinone) were collected at the moment of midodrine initiation and compiled alongside the cumulative vasopressor index, which reflects the additive effects and potency of various intravenous vasopressors by assigning cumulative points for equivalent doses of commonly used agents and has been described in other trials [33–35] (see Additional file 2: Table S1). Occurrence of kidney injury was noted as per the Kidney Disease: Improving Global Outcomes group (KDIGO) classification [36]. Severe acute kidney injury was defined as an increase of more than 100% in serum creatinine, serum creatinine ≥ 354 μmol/L with evidence of a minimum increase of > 26 μmol/L from baseline, or the initiation of dialysis during ICU stay (KDIGO stage ≥ 2). Intravenous vasopressor reinstitution was noted if initial discontinuation lasted at least two hours. ICU and hospital discharge were collected as the time the patient physically left the ICU or our institution and time spent at other hospitals was not considered. In-hospital mortality was collected and patients were considered alive at 30 days if they had been discharged alive from the hospital.

### Statistics

Dichotomous data were compared using χ2 tests or Fisher's exact test. Assumption of normality of data was evaluated with Shapiro–Wilk test. Continuous measurements were compared with t-test or the Mann–Whitney U-test, when appropriate, with calculation of difference and confidence interval between non-normally distributed variables calculated with the Hodges-Lehman method. To better assess the individual effect of midodrine on clinical outcomes, we used a linear regression model with Euroscore II, CPB duration, Propensity Score (PS) and midodrine as independent variables and ICU-free days as continuous dependant variable. We also performed multivariable logistic regressions with Euroscore II, CPB duration, PS and midodrine as independent variables and in-hospital mortality and ICU readmission as dichotomous dependent variables. Dependent and independent variables were selected a priori, based on clinical relevance, and were introduced into the model simultaneously. A Cox regression analysis was also performed, including the same independent variables, displaying survival at 30 days and time before cessation of IV vasopressors.

Data are presented as median and inter-quartile range [IQR] for non-normally distributed variables and as mean ± standard deviation for normally distributed variables. Comparative data is reported as odds ratios (ORs) or hazard ratios (HRs) with 95% confidence intervals. The analyses were performed with SPSS version 25 (IBM Corp., Armonk, NY, USA) with a statistical significance threshold of *p* < 0.05 without correction for multiple testing.

## Results

Within the study period, patients who had received midodrine in the ICU represented 2.1% of all patients who had underwent cardiac surgery with CPB. After PS matching, the groups demographic and intra-operative variables showed no statistical differences (Table 1). The final PS-matched sample consisted of 74 patients in each group.

Midodrine was initiated at a median time of 27.5 [21–49] hours after ICU admission, with 37 (50%) during the first 24 h and 70 (94.6%) during the first 72 h (see Additional file 3: Figure S2). Initial dose of midodrine was 10 mg in most patients (*N* = 61, 82.4%) and was rarely increased, as doses of more than 10 mg were prescribed in only two patients (2.7%). Midodrine was prescribed to be administered three times daily in all patients.

All patients were receiving norepinephrine infusion at the time of midodrine initiation, at a median dose of 0.05 [0.03–0.09] mcg/kg/min. Eleven patients (14.96%) were also receiving another intravenous vasopressor at that time (8 patients receiving norepinephrine and vasopressin, 2 patients receiving norepinephrine and epinephrine and 1 patient receiving all three). Cumulative vasopressor index was 3 [2–4] at midodrine initiation for the whole study group and inotropic support (milrinone or dobutamine) was underway in 6 patients (8.1%). Median midodrine treatment duration was 40 [23–73] hours and progressive tapering of midodrine doses had been done in 19 patients (26%).

Intravenous vasopressors were weaned off after a median of 19 [4–44] hours after the first dose of midodrine. In 16 (21.6%) patients, intravenous vasopressors had to be reinitiated more than two hours after initial cessation because of recurring hypotension despite midodrine. The total time on intravenous vasopressor was 63 [40–87] hours. In the midodrine group, the time to treatment initiation had a linear relation with the total time under intravenous vasopressor (R linear = 0.76, *p* < 0.001), whereas no correlation was found between the time to midodrine initiation and the time to intravenous vasopressors cessation after midodrine initiation (see Additional file 3: Figure S2). Seventeen patients (23%) were discharged from the ICU while still receiving midodrine and of these, only one patient (5.9%) was subsequently readmitted to the ICU and later died from septic shock. Of the other 57 patients who received their last dose of midodrine in the ICU, 9 (15.8%) eventually died and 5 (8.8%) were subsequently readmitted.

Comparative clinical outcomes are presented in Table 2. Compared to the control group, patients in the midodrine group presented significantly less days free from ICU at 30 days (25.8 [23.7–27.1] vs 27.2 [25.9–28] days, difference by Hodges-Lehman 1.7 days (CI 0.9–2.2), *p* = 0.002). The difference in ICU-free days was still significative after excluding patients who were readmitted to the ICU after initial discharge (25.9 [23.9–27.2] vs 27.2 [25.9–28.0] days, *p* = 0.004). Cumulative ICU length of stay was also higher in the midodrine group than in control group (99 [68–146] vs 68 [48–99] hours, *p* = 0.001). This difference was still significative after excluding readmitted patients (98 [68–144] vs 70 [49–100] hours, *p* = 0.009).

**Table 2:** Comparative clinical outcomes

Outcomes	Midodrine group	Control Group	OR (CI)	*p* value
Days free from ICU at 30 days	25.8 [23.7–27.1]	27.2 [25.9–28]	N/A	0.002*
In-hospital mortality	10 (13.5%)	1 (1.4%)	12.5 (1.5–105.2)*	0.036*
ICU readmission	6 (8.1%)	2 (2.7%)	3.1 (0.8–19.2)*	0.103*
Total time on IV vasopressors (h)	63 [40–86.5]	44 [26–66]	N/A	0.052*
ICU length of stay	99 [68–146]	68 [48–99]	N/A	0.001
Severe acute kidney injury	11 (14.9%)	10 (13.5%)	1.12 (0.4–2.8)	0.462

^*^Odds ratios and *p* values were computed after controlling for Euroscore II, cardiopulmonary bypass duration and propensity score, in binomial logistic, linear and Cox regression. Severe acute kidney injury: KDIGO ≥ 2

**Fig. 1:**
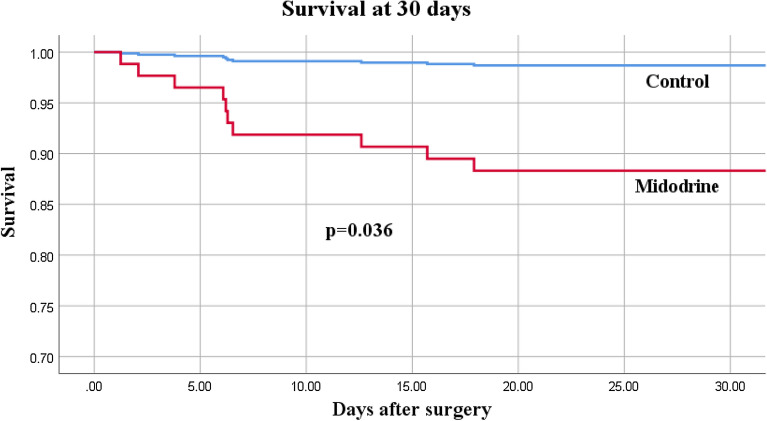
Survival at 30 days after cardiac surgery with Cox proportional hazard model after controlling for Euroscore II, CPB duration and propensity score. Adjusted Hazard Ratio for mortality with midodrine: 12.5 (1.5–105.2), *p* = 0.036

**Fig. 2:**
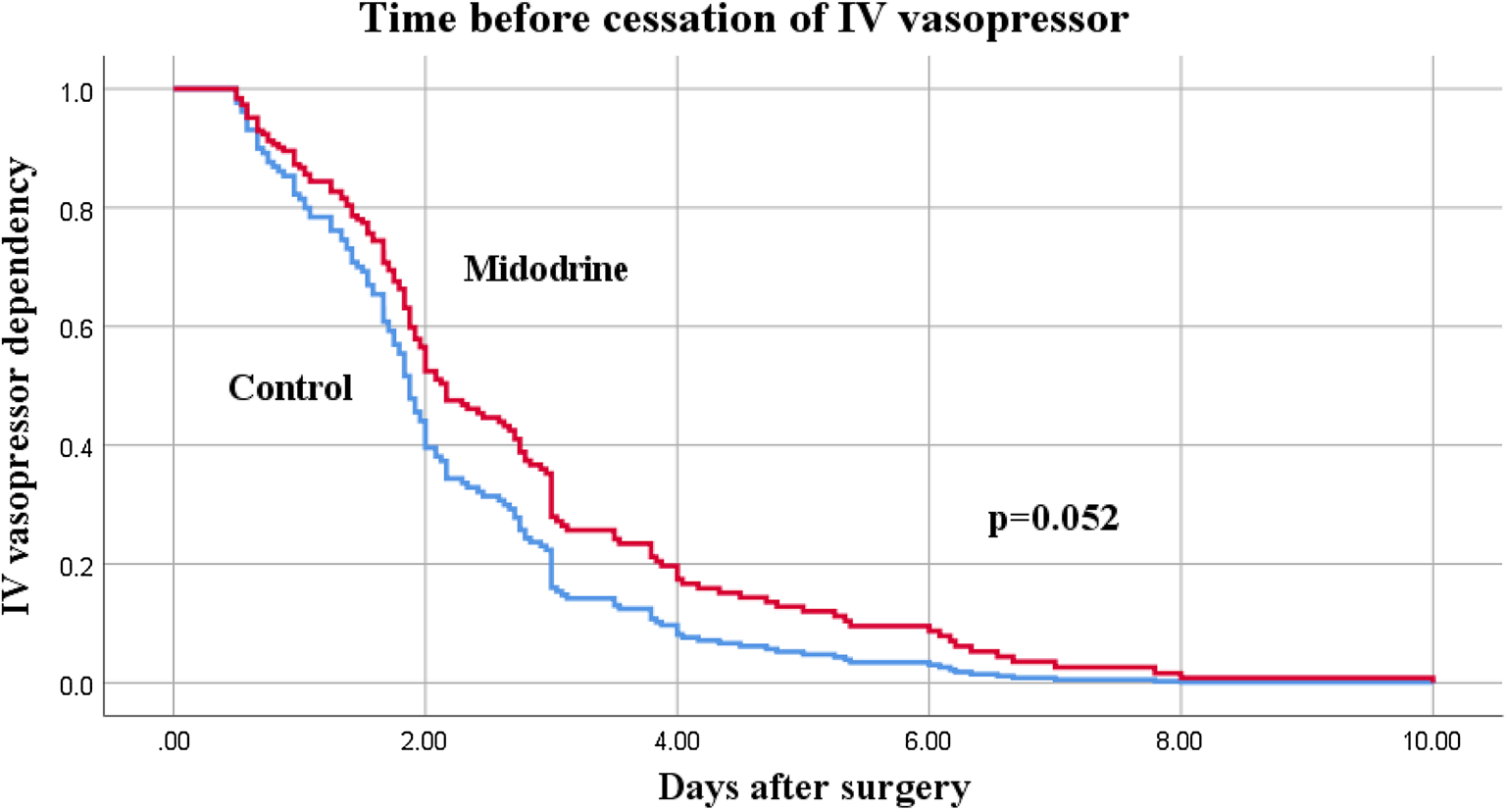
Cox regression depicting time (in days) before successful cessation of intravenous vasopressor between midodrine and control group after cardiac surgery, controlling for Euroscore II, CPB duration and propensity score. By design, patients in both groups had intravenous vasopressors for at least 12 h

Patients who received midodrine had a higher mortality rate than patients in the control group (10 (13.5%) vs 1 (1.4%), adjusted OR 12.5 (CI 1.5–105.2), *p* = 0.036, see Fig. 1) and tended to be readmitted more frequently to the ICU after initial discharge, although this did not reach statistical significance (6 (8.1%) vs 2 (2.7%), adjusted OR 3.1 (CI 0.8–19.2), *p* = 0.103). Of the 6 patients who were readmitted to the ICU in the midodrine group, 5 (6.8%) were readmitted for hemodynamic instability, namely tamponade (*n* = 2) sepsis (*n* = 1), persistent hypotension from vasodilation (*n* = 1) and unstable atrial fibrillation (*n* = 1). The 6 readmitted patients in the midodrine group spent a median of 53 [28–181] hours on the surgical ward before ICU readmission. The mortality rate in this group was 3 out of 6 (50%). Mortality was also high in patients receiving midodrine while under inotropic support (dobutamine or milrinone) in the ICU, with 3 out of these 6 patients who eventually died (50%). Overall, cases of death in the midodrine group included cardiogenic shock (*n* = 3), tamponade (*n* = 2), mesenteric ischemia (*n* = 2), septic shock (*n* = 2) and ventricular arrhythmia (*n* = 1). Total time on intravenous vasopressor was similar between groups, although it tended to be higher in the midodrine group than in control group (63 [40–87] vs 44 [26–66] hours, *p* = 0.052 by Cox regression, see Fig. 2).

## Discussion

This retrospective cohort study explored, for the first time, the clinical outcomes of patients who were prescribed midodrine after cardiac surgery with CPB in an effort to liberate them from intravenous vasopressors and hasten ICU discharge. In this specific group of patients, our data show that such a practice was associated with higher mortality rates and significantly less days free from ICU as compared with a matched contemporary control group with similar baseline characteristics.

In the last few years, an increasing number of authors have described the off-label use of midodrine for ICU patients with persistent hypotension attributed to loss of peripheral vascular resistance who are otherwise stable on small doses of intravenous vasopressors. Results from retrospective studies by Levine et al. [26] and Whitson et al. [25] suggested that midodrine in the ICU was associated with shorter time on intravenous vasopressors without significant side effects, although Poveromo et al. [27] have not been able to show such a benefit in a similarly designed study. More recently, results from a meta-analysis by Hammond et al. [29] did not show significant benefits of midodrine in accelerating weaning of vasopressors or reducing ICU length of stay or mortality. Of note, Rizvi et al. [23] have presented a mixed case series of 1119 patients who received midodrine during a 5 years period, showing a steady increase in the popularity of this practice in the ICU despite the absence of solid evidence in its favor, leading to heterogeneous and worrisome prescription patterns after ICU discharge [24]. A multicentric randomized controlled trial is underway to better assess the efficacy and safety of using midodrine in the ICU [28].

In our study, midodrine use was associated with significantly less days free from ICU and higher mortality. Although surprising, these findings need to be interpreted with caution, given the retrospective nature of our data and the inherent bias of indication. Still, the data suggest that up until midodrine initiation, these two groups presented somewhat similar baseline characteristics. Consequently, the reduction in ICU-free days and increase in mortality rate observed in the midodrine group cannot be simply attributed to difference in baseline characteristics, but may be due to other unknown residual confounders.

Nevertheless, multiple phenomena could explain that worrisome outcome. First, the hemodynamic effects of midodrine could be specifically deleterious in patients with subnormal ventricular function after cardiac surgery, as these patients could be more sensitive to increases in afterload in the absence of some inotropic support, potentially precipitating cardiac failure and hemodynamic collapse, as observed in three patients of the midodrine group. There is also a concern for mesenteric ischemia in these patients, as they often present baseline atherosclerosis and as such can suffer from impaired splanchnic arterial flow in the presence of arterial vasoconstriction with concomitant poor cardiac output, an outcome that was observed in two patients of the midodrine group. Kidney injury could potentially be precipitated by the same mechanism, although our results do not support such a claim, as the occurrence of severe kidney injury did not differ between groups and was somewhat comparable to similar cohorts, with reported rates between 9.0% and 44.4% [11, 37, 38]. Interestingly, such deleterious effects of arterial vasoconstriction without beta-adrenergic stimulation were one of the mechanisms that were put forward to explain the increase in mortality across the United States with norepinephrine shortage in 2011 and concomitant increased phenylephrine use [39].

Even though our results show that readmission rate alone does not explain the observed difference in mortality and ICU-free days, it might still be an important contributing factor. The readmission rate for patients who had received midodrine was abnormally high (8.1%), whereas in comparison, the control group presented a readmission rate of 2.7%, closer to our local overall rate of ICU readmission after cardiac surgery, which was just under 4% in 2018. By potentially facilitating liberation from intravenous vasopressors, midodrine might give a false impression of hemodynamic stability and could potentially obscure the first clinical clues of circulatory compromise, which could have especially dire consequences outside of the close monitoring provided by the ICU. In our cohort, 5 of the 6 readmitted patients presented significant hemodynamic instability. It is possible that appropriate interventions were delayed in such patients due to less intensive monitoring after ICU discharge.

Our study has significant limitations, namely due to its retrospective single center nature with small number of patients. As stated, confounding by indication likely contributes to the worse outcomes observed in the midodrine group, as the decision to initiate midodrine was made by the bedside clinician based on clinical characteristics or practice patterns that cannot be reliably captured and controlled in a retrospective study. Propensity score matching was performed to alleviate discrepancies between both groups, but only an eventual prospective randomized controlled trial will allow for proper control of confounding factors. In the meantime, considering the magnitude in outcome differences between groups, our results raise important safety concerns that should not be simply attributed to confounding by indication. Of note, our study design is prone to immortal time bias, but such a bias tends to underestimate bad outcomes in the study group [40], a phenomenon that cannot explain our findings.

## Conclusion

We report the practice of administering midodrine as an adjunct to intravenous vasopressors for persistent hypotension in an effort to accelerate ICU discharge after cardiac surgery. In this retrospective study, this approach was associated with fewer ICU-free days and higher mortality. This finding could be unique to post cardiac surgery patients, but caution should be encouraged in other clinical contexts. As this practice seems to be gaining in popularity, our results serve as a recall that trying to hasten ICU discharge should not be pursued at the expense of patients’ safety. Until further prospective studies are conducted, routine prescription of midodrine to accelerate ICU discharge after cardiac surgery should be done with caution.

## Supplementary information

**Additional file 1: Figure S1.** Distribution of the timing of Midodrine prescription after ICU admission (hours) of the entire Midodrine group, before the application of the inclusion criteria selecting patients with ≥ 12h hours of vasopressor (dotted line).

**Additional file 2: Table S1. **The cumulative vasopressor index.

**Additional file 3: Figure S2.** Correlation between the time to the first dose of Midodrine and the total time under vasopressors (*R* = 0.76, *p* < 0.001, Pearson) or between the time to the first dose of Midodrine and vasopressors weaning (*R* = 0.07, *p* = 0.96; Pearson).

## Data Availability

The datasets used in the study are available from the corresponding author on reasonable request.

## Abbreviations

ACE: Angiotension conversion enzyme
ARB: Angiotension receptor blocker
CPB: Cardiopulmonary bypass
ICU: Intensive care unit
PRBC: Packed red blood cells
