# Supplementary material for: Potential risks in using midodrine for persistent hypotension after cardiac surgery: a comparative cohort study
Source: Ann Intensive Care. 2020 Sep 14;10:121. doi: 10.1186/s13613-020-00737-w (PMC7490305; doi:10.1186/s13613-020-00737-w)
Supplement: Supplementary file 1 — Additional file 1: Figure S1. Distribution of the timing of Midodrine prescription after ICU admission (hours) of the entire Midodrine group, before the application of the inclusion criteria selecting patients with ≥ 12h hours of vasopressor (dotted line). [file 13613_2020_737_MOESM1_ESM.pptx]

## Slide 1
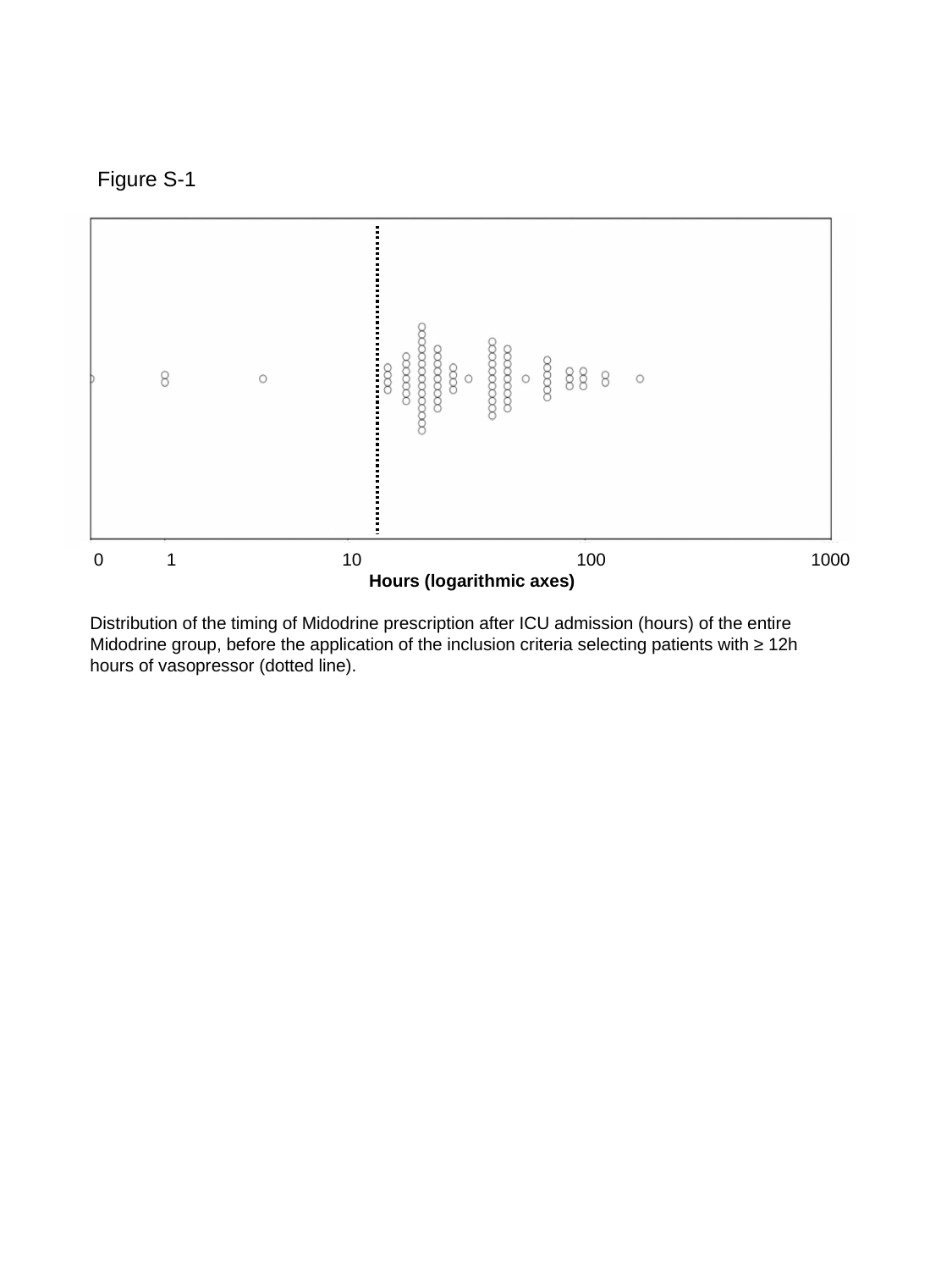

Figure S-1
0 1 	 10 	 100 	 1000
Hours (logarithmic axes)
Distribution of the timing of Midodrine prescription after ICU admission (hours) of the entire Midodrine group, before the application of the inclusion criteria selecting patients with ≥ 12h hours of vasopressor (dotted line).
