# Supplementary material for: Potential risks in using midodrine for persistent hypotension after cardiac surgery: a comparative cohort study
Source: Ann Intensive Care. 2020 Sep 14;10:121. doi: 10.1186/s13613-020-00737-w (PMC7490305; doi:10.1186/s13613-020-00737-w)
Supplement: Supplementary file 2 — Additional file 2: Table S1. The cumulative vasopressor index. [file 13613_2020_737_MOESM2_ESM.docx]

**Additional material**

Additional file 1: Table S1: The cumulative vasopressor index ^1^

| Vasopressor agent | Dose range 1 Point | Dose range 2 Points | Dose range 3 Points | Dose range 4 Points |
| --- | --- | --- | --- | --- |
| Dopamine (mcg/kg/min) | 0 - 5 | 5 - 10 | 10 - 15 | >15 |
| Epinephrine (mcg/kg/min) |  | 0 - 0.05 | 0.05 - 0.1 | >0.1 |
| Norepinephrine (mcg/kg/min) |  | 0 - 0.05 | 0.05 - 0.1 | >0.1 |
| Phenylephrine (mcg/kg/min) |  | 0 - 0.4 | 0.4 - 0.8 | >0.8 |
| Vasopressin (units/min) |  |  |  | any dose |

**1.** Trzeciak S, McCoy JV, Phillip Dellinger R, et al.: Early increases in microcirculatory perfusion during protocol-directed resuscitation are associated with reduced multi-organ failure at 24 h in patients with sepsis. Intensive care medicine*.* 34:2210-2217, 2008.
