# Supplementary material for: Potential risks in using midodrine for persistent hypotension after cardiac surgery: a comparative cohort study
Source: Ann Intensive Care. 2020 Sep 14;10:121. doi: 10.1186/s13613-020-00737-w (PMC7490305; doi:10.1186/s13613-020-00737-w)
Supplement: Supplementary file 3 — Additional file 3: Figure S2. Correlation between the time to the first dose of Midodrine and the total time under vasopressors (R = 0.76, p < 0.001, Pearson) or between the time to the first dose of Midodrine and vasopressors weaning (R = 0.07, p = 0.96; Pearson). [file 13613_2020_737_MOESM3_ESM.pptx]

## Slide 1
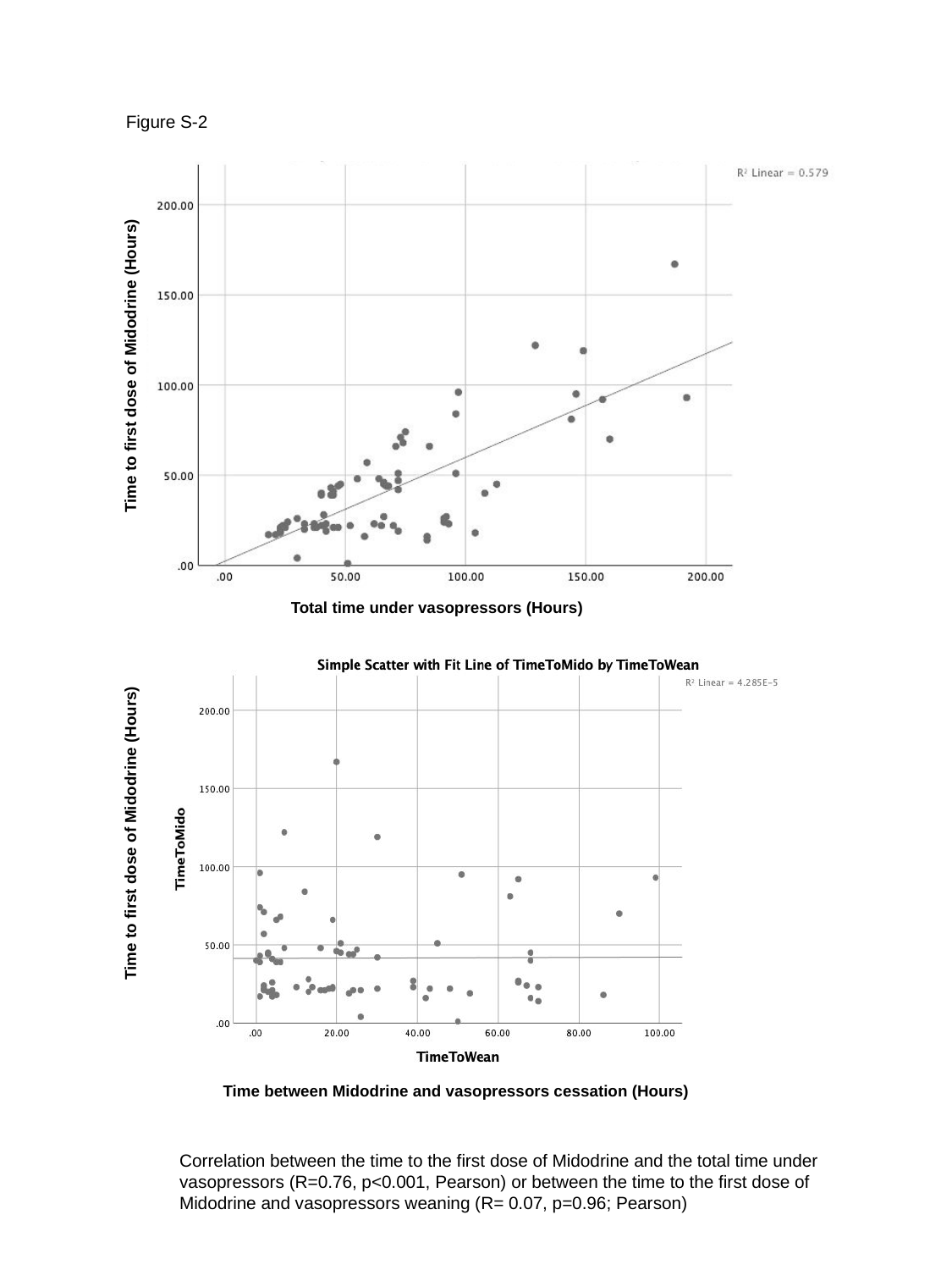

Figure S-2
Time to first dose of Midodrine (Hours)
Total time under vasopressors (Hours)
Time to first dose of Midodrine (Hours)
Time between Midodrine and vasopressors cessation (Hours)
Correlation between the time to the first dose of Midodrine and the total time under vasopressors (R=0.76, p<0.001, Pearson) or between the time to the first dose of Midodrine and vasopressors weaning (R= 0.07, p=0.96; Pearson)
